# Methylmercury Uptake into BeWo Cells Depends on LAT2-4F2hc, a System L Amino Acid Transporter

**DOI:** 10.3390/ijms18081730

**Published:** 2017-08-08

**Authors:** Christina Balthasar, Herbert Stangl, Raimund Widhalm, Sebastian Granitzer, Markus Hengstschläger, Claudia Gundacker

**Affiliations:** 1 Center for Pathobiochemistry and Genetics, Institute of Medical Genetics, Währinger Strasse 10, Medical University of Vienna, 1090 Wien, Vienna, Austria; christina.balthasar@gmail.com (C.B.); raimund.widhalm@meduniwien.ac.at (R.W.); Sebastian.Granitzer@kl.ac.at (S.G.); markus.hengstschlaeger@meduniwien.ac.at (M.H.); 2 Center for Pathobiochemistry and Genetics, Institute of Medical Chemistry, Währinger Strasse 10, Medical University of Vienna, 1090 Wien, Vienna, Austria; herbert.stangl@meduniwien.ac.at; 3 Karl Landsteiner University of Health Sciences, Dr.-Karl-Dorrek-Straße 30, 3500 Krems an der Donau, Austria

**Keywords:** methylmercury, leucine, methionine, human placenta, *SLC7A5*, *SLC7A8*, *SLC3A2*, Forskolin

## Abstract

The organic mercury compound methylmercury (MeHg) is able to target the fetal brain. However, the uptake of the toxicant into placental cells is incompletely understood. MeHg strongly binds to thiol-S containing molecules such as cysteine. This MeHg-l-cysteine exhibits some structural similarity to methionine. System L plays a crucial role in placental transport of essential amino acids such as leucine and methionine and thus has been assumed to also transport MeHg-l-cysteine across the placenta. The uptake of methylmercury and tritiated leucine and methionine into the choriocarcinoma cell line BeWo was examined using transwell assay and small interfering (si)RNA mediated gene knockdown. Upon the downregulation of large neutral amino acids transporter (LAT)2 and 4F2 cell-surface antigen heavy chain (4F2hc), respectively, the levels of [^3^H]leucine in BeWo cells are significantly reduced compared to controls treated with non-targeting siRNA (*p* < 0.05). The uptake of [^3^H]methionine was reduced upon LAT2 down-regulation as well as methylmercury uptake after 4F2hc silencing (*p* < 0.05, respectively). These findings suggest an important role of system L in the placental uptake of the metal. Comparing the cellular accumulation of mercury, leucine, and methionine, it can be assumed that (1) MeHg is transported through system L amino acid transporters and (2) system L is responsible for the uptake of amino acids and MeHg primarily at the apical membrane of the trophoblast. The findings together can explain why mercury in contrast to other heavy metals such as lead or cadmium is efficiently transported to fetal blood.

## 1. Introduction

One essential function of the human placenta is to accomplish the exchange of nutrients, gases, and metabolites between the mother and the fetus. The placental tissue is a barrier separating the maternal and fetal blood stream. Any substance that crosses the maternal-fetal interface from the maternal to the fetal side has to pass the outer syncytiotrophoblast (STB), the underlying cytotrophoblast (CTB), and the fetal endothelial cells (FECs). The initially complete cytotrophoblast layer becomes discontinuous as pregnancy progresses, resulting in just two continuous layers (STB and FECs) in the term placenta.

The human placenta is highly vulnerable to toxicants, including heavy metals [1,2,3]. It is evident that mercury crosses the placenta, accumulates in placental tissue, and passes onto the fetal blood and fetal organs [4,5,6]. An active transport across the human placenta has been assumed as cord blood mercury levels are, on average, almost twice the levels of maternal blood [7]. In a recent study, we showed system L transporters to be involved in methylmercury uptake into human placental cells, i.e., human primary trophoblasts and the human choriocarcinoma cell line BeWo [8].

The organic mercury compound methylmercury (MeHg) is a toxicant well known to target neurodevelopment. The toxicokinetics of mercury are determined by its high affinity to sulfhydryl groups. Methylmercury is present in the body as water-soluble complexes, mainly, if not exclusively, attached to the sulphur atom of thiol ligands such as l-cysteine, glutathione, or metallothioneins [9]. MeHg-l-cysteine, at least its amino acid component, is recognized by system L transporters [10]. These are heterodimers comprised of a light chain (large neutral amino acids transporter, LAT1 or LAT2) covalently bound by a disulfide bridge to a heavy subunit (4F2 cell-surface antigen heavy chain, 4F2hc) (syn. CD98). The solute carriers LAT1-4F2hc (*SLC7A5-SLC3A2*) and LAT2-4F2hc (*SLC7A8-SLC3A2*) are obligatory exchangers (1:1 stoichiometry), transporting large branched and aromatic neutral amino acids, including leucine and methionine. LAT2 also transports some smaller amino acids. The tissue distribution and cellular localization of LAT1 suggests that its main role is to transport amino acids into proliferating cells and across some barriers, including the placenta, while LAT2 is rather involved in the efflux step of transepithelial amino acid transport [11,12]. Previous studies indicate that these amino acid transporters also transport methylmercury across membranes of various cell types [13,14,15,16] and the rat placenta [17].

We aimed to analyse the uptake of methylmercury into placental cells in a setting as close as possible to the in vivo situation. Polarized cells take up and efflux molecules through both their basal and apical surfaces when grown on transwell supports. BeWo cells, notably the clone 24, build confluent, polarized monolayers, forming tight-junctions and microvilli on the apical side [18], enabling the study of the uptake, intracellular transport, and release of molecules on both their basal and apical membranes. In addition, the trophoblast-derived cells still display characteristics of human primary trophoblast cells, including the secretion of human chorionic gonadotropin (hCG). BeWo cells are therefore widely used to study that transport of nutrients, drugs, pathogens, and immunoglobulins across the STB [19].

The study goals were: (1) to establish the role of LAT1, LAT2 and 4F2hc in MeHg uptake into BeWo cells by comparing MeHg uptake with uptake of [^3^H]methionine and [^3^H]leucine into BeWo cells upon LAT1, LAT2 and 4F2hc silencing, respectively, and (2) to determine flux of the substrates in relation to transporter localization.

## 2. Results

### 2.1. Pre-Experiments

**Figure 1:**
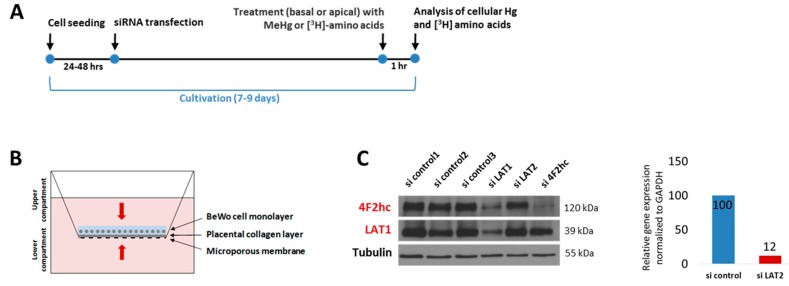
Experimental protocol and knockdown efficiency. (**A**) siRNA-mediated gene knockdown was conducted between 24 h and 48 h after seeding. BeWo cells were exposed to 2 µM MeHg or [^3^H] amino acids around day 8 for one hour. To control for monolayer permeability, Lucifer Yellow (experiments on MeHg) and [^14^C]mannitol (experiments on amino acids) were used (**B**) Transwell assay to study the uptake of MeHg and [^3^H] amino acids into BeWo cells. (**C**) Confirmation of knockdown efficiency on protein level by immunoblotting (LAT1, 4F2hc) and on mRNA level by qPCR (LAT2) from one representative experiment. All three commercially available LAT2 antibodies (Table 1) were proven unable to detect the protein [20].

The BeWo cell culture protocol was optimized to study the uptake of MeHg, [^3^H]methionine, and [^3^H]leucine into placental cells in transwell plates (Figure 1A,B). Immunoblots (LAT1 and 4F2hc) and quantitative polymerase chain reaction (qPCR) (LAT2) confirmed efficient siRNA mediated knockdown under these conditions (Figure 1C). It has to be noted that, upon LAT1 downregulation, 4F2hc expression is also markedly reduced.

**Figure 2:**
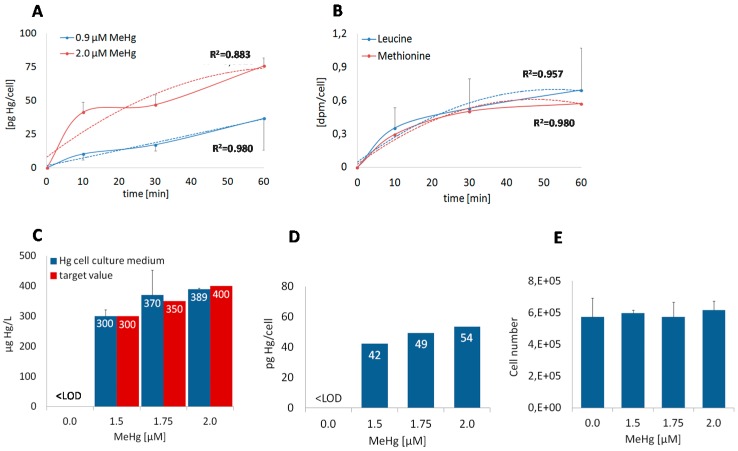
Time-dependent uptake of MeHg, [^3^H]methionine, and [^3^H]leucine. (**A**) BeWo cells were treated with 0.9 µM and 2 µM MeHg and (**B**) with 1 µCi/mL [^3^H]leucine (100 Ci/ mmol) and 1 µCi/mL [^3^H]methionine (84.5 mCi/mmol) for 10 min, 30 min, and 60 min in transwell plates. Data represent mean values ± SD from two independent experiments. **(C)** The apical addition of 0.0, 1.5 µM, 1.75 µM, and 2.0 µM MeHg (target values in red) to cell culture medium leads to the dose-dependent increase of total mercury concentrations in BeWo cells (**D**) after one hour at (**E**) unchanged cell numbers. (**C**, **E**) The data represent mean values ± SD from one experiment made in triplicate. (**D**) Prior to mercury analysis, BeWo cell aliquots were pooled.

First we conducted a time course experiment to compare the uptake of MeHg and the amino acids; BeWo cells were exposed to two MeHg doses (0.9 µM, 2.0 µM) and to 1 µCi/mL tritium-labeled methionine and leucine, respectively, for 10 min, 30 min, and 60 min (Figure 2A,B). The 0.9 µM dose was selected as a multiple of 0.03 µM MeHg, which is equivalent to about 6 µg/L, representing physiological concentration [8]. As expected, mercury levels were twofold higher in cells exposed to 2.0 µM MeHg than in those exposed to 0.90 µM MeHg. Compared to the rather continuous uptake of amino acids, the uptake of methylmercury, particularly the 2 µM dosage, occurred in a non-linear manner. It was strongest during the first ten minutes, followed by a steady uptake for further 20 min, and then uptake increased significantly again. The incubation time for all following experiments was set at 60 min. At that time point, the cells had accumulated detectable concentrations of all substrates (Figure 2A,B). As shown in Figure 2C,D, BeWo cells exposed to 2 µM MeHg (i.e., 180 µg/L) for one hour accumulate mercury to non-cytotoxic levels as far as indicated by unchanged cell numbers (Figure 2E).

### 2.2. Forskolin-Induced BeWo Cell Fusion Increases LAT1 and LAT2 Expression

**Figure 3:**
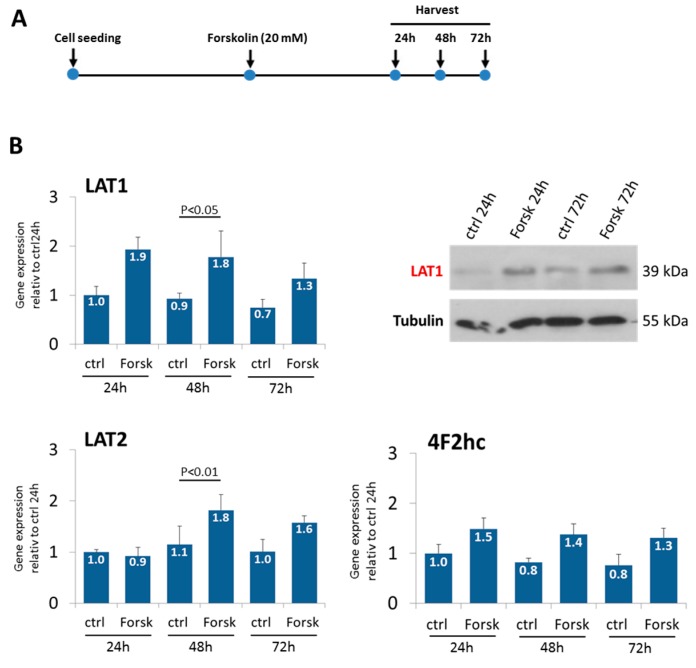
Expression of LAT1, LAT2, and 4F2hc under Forskolin treatment. (**A**) BeWo cells were treated with 20 mM Forskolin when about 50% confluent and harvested after 24, 48, and 72 h. (**B**) Gene expression of LAT1, LAT2, and 4F2hc over time. Increased LAT1 levels upon Forskolin treatment were confirmed via immunoblotting. One representative Western Blot is shown. Data are mean values ± SD from one experiment based on four replicates. Results from ANOVA are given when *p* < 0.05. ctrl: Control; Forsk: Forskolin.

The Forskolin-induced fusion of BeWo cells was used to analyze changes of system L expression during differentiation (Figure 3A). Both LAT1 and LAT2 expression significantly increased upon 48 h of Forskolin treatment in relation to dimethyl sulfoxide (DMSO) treated controls, an effect confirmed by immunoblotting for LAT1 (Figure 3B).

### 2.3. LAT2 and 4F2hc Downregulation Reduces Mercury Uptake into BeWo Cells

**Figure 4:**
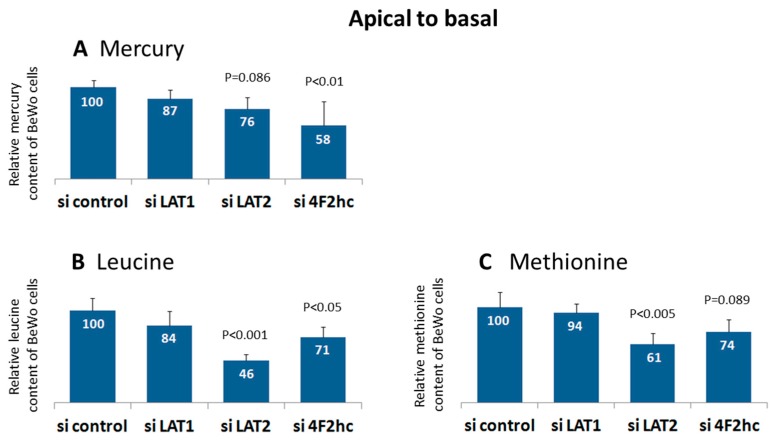
Uptake of MeHg, [^3^H]methionine, and [^3^H]leucine upon system L subunit silencing. Relative cellular contents of (**A**) total mercury, (**B**) [^3^H]leucine, and (**C**) [^3^H]methionine in BeWo cells after LAT1, LAT2, and 4F2hc silencing. MeHg, [^3^H]leucine, and [^3^H]methionine were added to the apical chamber of the transwell. Data are mean values ± SD from three independent experiments; results from ANOVA are given when *p* < 0.1.

Adding MeHg to apical compartments upon LAT2 and 4F2hc silencing resulted in significantly decreased mercury content of the BeWo cells (76% and 58%, respectively) in relation to the controls (Figure 4A). No such effect could be detected when methylmercury was added to the basal compartment (data not shown). The basal to apical permeability determined by Lucifer Yellow paracellular transport was 5.2 ± 1.7% (*n* = 8) and was approximately twice as high as that from apical to basal (3.4 ± 1.3%, *n* = 8). The ratio thus, as expected, correlated to the ratio of basal to apical volumes of media (2:1).

### 2.4. LAT2 and 4F2hc Downregulation Reduces Methionine and Leucine Uptake into BeWo Cells

LAT2 and 4F2hc downregulation resulted in the significantly reduced uptake of leucine (46% and 71%, respectively) and methionine (61% and 74%, respectively) when amino acids were added to the apical chamber (Figure 4B,C). No such effect was seen when the amino acids were added to the basal compartment (data not shown). In LAT1 downregulated BeWo cells, a trend for lower leucine uptake was observed. The permeability determined by paracellular mannitol transport was 2.1 ± 0.5% (*n* = 6) in experiments examining apical to basal leucine transport. The basal to apical permeability was 4.5 ± 0.9%. With regard to methionine transport, apical to basal permeability was 2.2 ± 0.4% (*n* = 6), and basal to apical permeability was 5.0 ± 1.3% (*n* = 6). The ratio of permeability thus, as expected, correlated with the ratio from apical to basal volumes of media (1:2).

## 3. Discussion

The concept of a placenta barrier suggests that a placental cell, first and foremost the STB, is able to distinguish between essential nutrients that have to be transported to the fetal blood stream and unwanted substances that should not reach the fetal circulation. It is, however, evident that the toxicant mercury in the form of MeHg-l-cysteine is recognized by system L when expressed at the blood brain barrier or in *Xenopus laevis* eggs (e.g., [13,14]). The question arose whether the toxicant is transported in the same way as amino acids across the human placenta.

While placental amino acid transport is comparatively well understood [11,21], our knowledge on placental mercury transport still is incomplete. The aim of the present study was to address the role of placental system L amino acid transporters in MeHg uptake into BeWo cells, a trophoblast transport model endogenously expressing system L. It has to be noted that BeWo cells are mostly mononuclear (if not stimulated to fuse in vitro) and thereby model the undifferentiated trophoblast rather than the syncytiotrophoblast. As human primary trophoblast cells start to differentiate rapidly after plating and form syncytia in a discontinuous manner [22], they are rarely used in transwell studies. However, in a recent report, a validated model of a confluent human primary trophoblast monolayer has been proposed [23].

Previous findings [8,9,13,14,24,25,26] suggest that MeHg transport across barriers depends on cysteine, is stereo-selective (MeHg is transported in presence of l-cysteine but not in presence of d-cysteine), and is carrier-mediated by system L and system b^0,+^. In vitro demethylation to mercuric mercury is implausible as, in humans, MeHg is slowly metabolized to inorganic mercury, predominantly by the intestinal microflora at a rate of about 1% of the body burden per day and to some extent also in phagocytic cells. It is therefore to be expected that most of the MeHg added to cell culture medium is present as monovalent cation (CH_3_Hg^+^) rapidly bound to ligands due to the high affinity of mercury ions to sulfhydryl group-containing molecules. Although it is likely that a substantial part of MeHg is bound to cysteine in the extracellular space as well as to glutathione in the cytosol, the respective amounts of MeHg compounds present in the extra- and intracellular compartments have not been quantified so far. It has to be noted that the amino acid composition of the serum (FCS) we added to cell culture medium is not provided by the manufacturer.

In order to demonstrate the inhibition of MeHg transport after the inactivation of system L subunits, we studied the transport of methionine, leucine, and methylmercury in parallel. Methionine was included because MeHg-l-cysteine structurally mimics the amino acid part of the molecule [10], while leucine specifically reflects system L activity as the amino acid is mainly transported by system L [11]. In this work, we provide evidence that MeHg is transported across BeWo cells through system L amino acid transporters.

### 3.1. Dose and Time Dependent Uptake into BeWo Cells

BeWo cells accumulate mercury to levels directly proportional to MeHg dosages. The mercury levels do not reach equilibrium during the first hour of exposure. Primary human trophoblast cells reach a steady state in mercury accumulation after about four hours [8]. Both methionine and leucine reach a steady state at around 30 to 60 min. The findings are in accordance with the time course of histidine uptake through LAT1 [27]. Simmons-Willis et al. [14] suggested MeHg-cysteine to be a better substrate for system L than endogenous amino acids, as they observed higher V_max_ values for MeHg-l-cysteine than for methionine transport through LAT1 and LAT2. Nonetheless, this finding cannot explain why BeWo cells still accumulate mercury while amino acid levels are already in a steady state. System L activity in combination with other amino acid uniporters/exchangers obviously tightly regulates intracellular methionine and leucine levels, while MeHg, once in the cell, dissociates from cysteine to bind to other intracellular ligands, e.g., glutathione or metallothionein [28], and thus no longer is under the control of amino acid transporters.

### 3.2. LAT1, LAT2, and 4F2hc Downregulation Does Not Affect BeWo Cell Number

The validation of siRNA-mediated gene knockdown by immunoblotting showed that the silencing of LAT1 results in the downregulation of 4F2hc. LAT2 knockdown could be confirmed on the mRNA level as commercial LAT2 antibodies (Table 1) were shown to be unable to detect the target protein [20].

We observed BeWo cell numbers to remain unaffected by the downregulation of any of the system L subunits (data not shown). In addition, no effects on cell morphology such as shrinking could be observed by visual inspection (inverted light microscope). Amino acid supply is crucial for cell growth and proliferation [29]. Gene targeting of *Slc3a2* (4F2hc) in conventional knockout mice is embryonically lethal as it is obligatory for murine embryogenesis [30]. 4F2hc was shown to play a role in tumorigenesis in renal cancer cell lines [31] and in the skin homeostasis of *Slc3a2* conditional knockout mice [32]. A global homozygous knockout of *Slc7a5* (LAT1) in mice was also embryonically lethal. The heterozygous *Slc7a5* knockout animals, however, had no overt phenotype, suggesting that its function in mTOR-S6K signaling is sufficiently compensated by, for instance, LAT2 [33]. A loss of LAT1 results in tumor growth inhibition [34]. In contrast to 4F2hc and LAT1, the *Slc7a8* (LAT2) knockout mouse did not apparently differ from the wild type mouse, apart from a mild aminoaciduria [35]. Overall, we conclude that our experimental model (i.e., a transfection period of five to seven days; Figure 1A) does not mimic the long-term effects of system L subunit inhibition.

### 3.3. Forskolin-Induced BeWo Cell Fusion Increases LAT1 and LAT2 Expression

Our observation that Forskolin induces the up-regulation of LAT1 is in accordance with previous reports in BeWo cells [36]. Elevated levels of LAT1 might be essential for the formation of the syncytiotrophoblast since a recent study has demonstrated impairments in trophoblastic fusion in LAT1 knockdown mice as well as in LAT1 deficient BeWo cells [37].

### 3.4. LAT2 and 4F2hc Downregulation Reduces Uptake of Methylmercury, Leucine and Methionine into BeWo Cells

BeWo cells accumulate significantly less mercury (76% and 58%) upon LAT2 and 4F2hc silencing relative to controls. This finding is, in principle, in accordance with other reports showing the transporter subunits to be involved in MeHg uptake into rat brain [13], C6 rat glioma cells [38], B35 rat neurons [16], rat placenta [17], *Xenopus laevis* oocytes [14], and Chinese hamster ovary cells [15]. In our previous study on BeWo cells cultivated in conventional dishes, we observed a similar reduction of mercury uptake upon LAT2 silencing (75%) but no such effect upon 4F2hc silencing. Moreover, we found the strongest effect on cellular mercury when LAT1 was down-regulated [8]. It remains unclear whether these discrepancies emerge from the different methods of cell culturing (conventional dishes versus transwell), leading to differences in cell morphology and polarity [39], or from the different MeHg treatments (0.90 µM in our previous work versus 2 µM in the present study).

BeWo cells responded to the down-regulation of LAT2 and 4F2hc with significantly reduced uptake of methionine (61% and 74%) and leucine (46% and 71%), whereas LAT1 silencing had no apparent effect (94% and 84%). The latter observation is in accordance with a previous report from Gaccioli et al. [40] in human primary trophoblast cells. The less pronounced effect of LAT2 knockdown on leucine uptake in human primary trophoblast cells (uptake reduced to 87% relative to controls) compared to BeWo cells (reduced to 46%; Figure 4B) might be explained by the circumstance that human primary trophoblasts are hard to transfect.

In transwell experiments with BeWo cells, LAT1 knockdown had no significant effect on that transport of methylmercury, leucine, and methionine, although we observed a trend for lowered uptake here as well (Figure 4). In *X. laevis* oocytes, LAT1 was shown to transport the substrate faster than LAT2 (*V*_max_ of 286 vs.75) but also to have a lower affinity to MeHg-l-cysteine than LAT2 (*K*_m_ of 98 µM vs. 64 µM) [14]. To our knowledge, no other studies exist in which mercury and amino acid uptake have been directly compared. In lung cancer cells, Dann et al. [34] observed a significant reduction of methionine levels (to about a third relative to controls) upon the downregulation of LAT1. Nicklin et al. [29] found both LAT1 and 4F2hc silencing to exert the same effect on leucine transport (reduction to a third) into HeLa cells.

### 3.5. Uptake of MeHg, Leucine and Methionine in Relation to Transporter Localization

Most of the so far available data suggest that system L transporters, LAT1, LAT2, and 4F2hc, respectively, localize primarily to the apical side of the STB [41,42,43]. Our data based on RNAi validated antibodies [20] showed the heavy chain 4F2hc to be localized at both STB plasma membranes (apical and basolateral). Moreover, we found the light chains, LAT1 and particularly LAT2, localized in intracellular vesicular structures of the STB [8]. Our data indicate that LAT2-4F2hc exerts its function predominantly at the apical side of trophoblast cells, as the uptake of mercury, leucine, and methionine remained unaffected by system L inactivation when substrates were added to the basal chamber.

## 4. Materials and Methods

### 4.1. Cell Culture

BeWo cells (clone 24), a kind gift from Dr. Isabella Ellinger (Medical University of Vienna), were cultured at 37 °C and 95% air/5% CO_2_ in DMEM high glucose (Life Technologies, Carlsbad, CA, USA), supplemented with 10% FBS Good (Pan Biotech, Aidenbach, Germany) and 1% GlutaMAX (Life Technologies). The cells were detached from culture dishes with trypsin/ethylenediaminetetraacetic acid (EDTA). The cell number was determined with a CASY cell counter and analyzer (CASY^®^ Model TTC 45/60/150, Innovatis Technologies Inc., Woodbridge, VA, USA).

### 4.2. Transwell Studies

BeWo cells were seeded onto permeable Transwell inserts (12-well polycarbonate membrane with 0.4 µm pore size, Corning Inc., Corning, NY, USA) that had been coated with human placental collagen (50 µg/cm²; Bornstein and Traub Type IV, Sigma-Aldrich Corporation, St. Louis, MO, USA) according to the protocol of Bode et al. [22] at a density of 2.5 × 10^4^ cells/well. The cells were transiently transfected 24 h to 48 h post seeding with non-targeting and specific siRNA targeting *SLC7A5*, *SLC7A8*, and *SLC3A2* (encoding LAT1, LAT2, and 4F2hc) (GE Dharmacon, Lafayette, CO, USA) using Lipofectamine RNAiMax (Life Technologies) as described by Rosner et al. [44], with the minor modification of employing only ¼ of the original transfection reagent amount. Thereafter the cells were cultivated until a confluent monolayer was formed (around eight days after seeding).

BeWo cells were treated with 2 µM MeHg (aqueous CH_3_HgCl) (Alfa Aesar, Haverhill, MA, USA) for one hour, added to medium at the apical (0.5 mL) or basal (1 mL) side of the transwell. The paracellular permeability of each well was determined concomitantly to MeHg transport by adding 100 µM Lucifer Yellow (CH Dilithium Salt, Sigma-Aldrich) to the target compartment, while the opposing compartment was filled with cell culture medium only. Lucifer Yellow’s fluorescence was measured in black 96-well plates (Corning) in a microplate reader (BioTek Instruments, Winooski, VT, USA) using a 485 ± 20 nm excitation and 528 ± 20 nm emission filter.

In the same way as for MeHg, BeWo cells were treated with 1 µCi/mL [^3^H]Leucine (100 Ci/mmol, Hartmann Analytik, Braunschweig, Germany) or 1 µCi/mL [^3^H]Methionine (84.5 mCi/mmol, Perkin Elmer Inc., Waltham, MA, USA) in Hank’s Balanced Salt Solution (HBSS) (Sigma-Aldrich), supplemented with 0.2 µCi/mL [^14^C]mannitol (56.8 mCi/mmol, Perkin Elmer) to test for cell permeability. After incubation, the cells were washed three times with ice cold Hank’s balanced salt solution (HBSS), followed by lysis in 250 µL NaOH (1 mol/L, Merck & Co., Kenilworth, NJ, USA). 200 µL of solubilized cells were added to 5 mL liquid scintillation fluid (Ultima Gold, Perkin Elmer), and the radioactivity of each cell lysate sample was determined by scintillation counting (TRI-Carb 2800 TR, Perkin Elmer).

### 4.3. Forskolin Treatment

BeWo cells were cultured until 50% confluency on 60 mm dishes (Corning). At this point, the cells were either incubated with Forskolin (20 mM) or DMSO as a control. Cells were harvested after 24 h, 48 h, and 72 h. Changes in gene expression were analysed by quantitative PCR (qPCR) and Immunoblotting.

### 4.4. RNA Isolation, cDNA Synthesis and Quantitative PCR

Total RNA was isolated using TRI Reagent® (Sigma), according to the manufacturer’s instructions. RNA was reverse transcribed with a Go-Script Reverse Transcription System (Promega, Madison, WI, USA) using random hexamer primers. Gene expression was analyzed using a Taq Man Expression System (Applied Biosystems, Foster City, CA, USA) in an Applied Biosystems StepOnePlus™ Real-Time PCR System, according to the manufacturer’s protocol. The cDNA was diluted 1:11, and 2 µL was used as a template in a 15 µL reaction. Glyceraldehyde 3-phosphate dehydrogenase (GAPDH) and TATA-box binding protein (TBP) were used as reference genes. The employed primers were Hs00794796 m1 (SLC7A8), Hs99999905_m1 (GAPDH), and Hs00427620_m1 (TBP). In Forskolin experiments, we used the primers Hs00185826_1 (SLC7A5), Hs00247916_m1 (LAT2), and Hs00374243_m1 (SLC3A2), and as reference gene we used Hs0082473_m1 (Ubiquitin C).

### 4.5. Protein Extraction and Immunoblotting

The cells were lysed in RIPA (Radioimmunoprecipitation assay) buffer (50 mM Tris, pH 7.6, 150 mM NaCl, 1% Triton, 0.1% SDS, 0.5% sodium deoxycolate), supplemented with 2 mg/mL aprotinin, 0.3 mg/mL benzamidin chloride, 2 mg/mL leupeptin, and 10 mg/mL trypsin inhibitor (Sigma). The protein samples were separated using SDS-PAGE and transferred to nitrocellulose membranes. Blots were blocked for 1 h in 5% nonfat dry milk in tris-buffered saline containing 0.1% Tween 20 (TBST), followed by incubation in 5% bovine serum albumin (BSA)/TBST containing the primary antibody overnight at 4 °C. Thereafter, blots were washed and incubated with corresponding secondary horseradish peroxidase (HRP)-conjugated antibodies. The enhanced chemiluminescence method (Pierce™ ECL western blotting substrate, Thermo Fisher Scientific, Waltham, MA, USA) was used to visualize the signals. For a list of the employed primary and secondary antibodies, see Table 1.

### 4.6. Analysis of Mercury

The samples and reference material were acid-digested with nitric acid (69%; Suprapur®; Carl Roth, Karlsruhe, Germany) in a microwave oven (MARS6, CEM Corporation, Matthews, NC, USA). The samples, stabilized with HCl, were stored at 4 °C for up to three days and diluted in a ratio of 1:2.5 before they were analyzed for total mercury content by cold vapour atomic fluorescence spectroscopy (CV-AFS) (Mercur Plus, Analytik Jena AG, Jena, Germany). Quality control was achieved by measuring blank test solutions (limit of detection was 0.024 µg/L) and reference materials (Seronorm Trace Elements Urine L-2, 210705, LOT 1011645). The mercury levels of the reference material (30.6 ± 7.2 µg/L; *n* = 19) lay well within the certified range (23.8–55.8 µg/L). All samples were measured in duplicate by the working curve method (RSD < 15%).

### 4.7. Statistics and Software

Data represent mean values ± SD (standard deviation). Regression lines and coefficients of determination were made with MS Excel. ANOVA was applied for the comparison of group differences, followed by a Bonferroni test to correct for multiple testing. We used IBM SPSS Statistics 24 (IBM, Armonk, NY, USA) and set the critical significance level at *α* = 0.05.

## 5. Conclusions

The present study is the first one in which uptake of methylmercury, leucine, and methionine were examined upon the knockdown of system L subunits LAT1, LAT2, and 4F2hc in parallel and in a setting as close as possible to the in vivo situation. The direction and magnitude of the effects are comparable. Altogether, the findings clearly indicate that LAT2-4F2hc is a significant contributor to methylmercury uptake into placental cells. The findings support the assumption that methylmercury (in the extracellular environment most likely present as MeHg-l-cysteine) is accidentally taken up into the human cytotrophoblast because the compound resembles essential amino acids. The ‘mimicry’ can explain why mercury in contrast to other heavy metals such as lead or cadmium is efficiently transported to the fetal blood.

## Figures and Tables

**Table 1:** List of the primary and secondary antibodies used in immunoblotting.

Product Name	Company, Article No. (Dilution)
anti-4F2hc rabbit polyclonal antibody	Cell Signaling, #13180 (1:1000)
anti-LAT1 rabbit polyclonal antibody	Cell Signaling, #5347 (1:1000)
anti-LAT2 mouse monoclonal antibody	OriGene, TA500513S (1:500)
anti-LAT2 rabbit polyclonal antibody	Santa Cruz, sc-133726 (1:100)
anti-LAT2 rabbit polyclonal antibody	ImmunoGlobe, 0142-10 (1:1000)
anti-α-Tubulin mouse monoclonal antibody	Merck, 05-829 (1:5000)
mouse IgG-heavy and light chain antibody	Bethyl, A90-116P (1:10,000)
rabbit IgG-heavy and light chain antibody	Bethyl, A120-101P (1:10,000)

Cell Signaling Technology: Danvers, MA, USA; OriGene: Rockville, ML, USA; Santa Cruz Biotechnology: Dallas, TX, USA; ImmunoGlobe: Himmelstadt, Germany; Bethyl: Montgomery, TX, USA.
